# Supplementary material for: BNT162b2 mRNA vaccine elicited antibody response in blood and milk of breastfeeding women
Source: Nat Commun. 2021 Oct 28;12:6222. doi: 10.1038/s41467-021-26507-1 (PMC8553805; doi:10.1038/s41467-021-26507-1)

BNT162b2 mRNA vaccine elicited antibody response in blood and milk of breastfeeding women

Michal Rosenberg Friedman[1,2], Aya Kigel[3], Yael Bahar[3], Michal Werbner[4], Joel Alter[5], Yariv Yogev[1,2], Yael Dror[3], Ronit Lubetzky[2, 6], Moshe Dessau[5], Meital Gal-Tanamy[4], Ariel Many[1,2] & Yariv Wine[3*]

[1]Department of Obstetrics and Gynecology, Lis Maternity & Women's Hospital, Tel Aviv Sourasky Medical Center, Tel Aviv, Israel

[2]Sackler Faculty of Medicine, Tel Aviv University, Tel Aviv, Israel

[3]The Shmunis School of Biomedicine and Cancer Research, The George S. Wise Faculty of Life Sciences, Tel Aviv University, Tel Aviv, Israel

[4]Molecular Virology Lab, The Azrieli Faculty of Medicine, Bar-Ilan University, Safed, Israel

[5]The Laboratory of Structural Biology of Infectious Diseases, The Azrieli Faculty of Medicine, Bar-Ilan University, Safed, Israel

[6]Department of Pediatrics, Dana Dwek Children's Hospital, Tel Aviv Sourasky Medical Center, Tel Aviv, Israel

Supplementary Table 1

Supplementary Figures 1-7

Supplementary Table 1 Study cohort information. Mean age 34.6 (range 30-38). Days from giving birth to first dose of BNT162b2 mRNA vaccine - mean 154 days (range 68-382)

Patient	Gravidity Parity (GP)	Gesta-tional age at delivery	From birth to first dose of BNT162b2 mRNA vaccine (days)	Infant nutrition	From TdaP vaccine to day 7 (months)	From last COVID-19 RT-PCR to first dose of vaccine (weeks)
MC1	G3P3	39+5	382	MOM, solid food	13	6
MC2	G5P3	39+0	109	MOM	6	2
MC3	G1P1	38+4	164	MOM, solid food	7	2
MC4	G3P2	37+6	68	MOM + formula	4	10
MC6	G2P1	40+6	152	MOM + formula	6	14
MC7	G4P3	40+0	148	MOM, solid food	8	21
MC8	G4P2	38+5	81	MOM	4	11
MC9	G2P2	40+0	143	MOM	6	0.5
MC10	G3P3	40+0	135	MOM, solid food	6	19
MC11	G2P2	38+4	159	MOM, solid food	7	23

* No infant co-morbidities.

MOM – mother's own milk.

Supplementary Fig. 1 Serial dilution ELISA to determine endpoint titers. Each plotted graph shows obtained O.D.$_{450}$ values that were subtracted from blank values for each plate. ELISA graphs pertain to serum or breastmilk samples obtained at 4 time points (by color) for each vaccinee and a pre-pandemic negative control that were tested against the SARS-CoV-2 **a** spike, and **b** RBD protein. Dashed red lines represent the threshold value equivalent to the background + 3 s.d. values. Each sample was measured in duplicates.

a

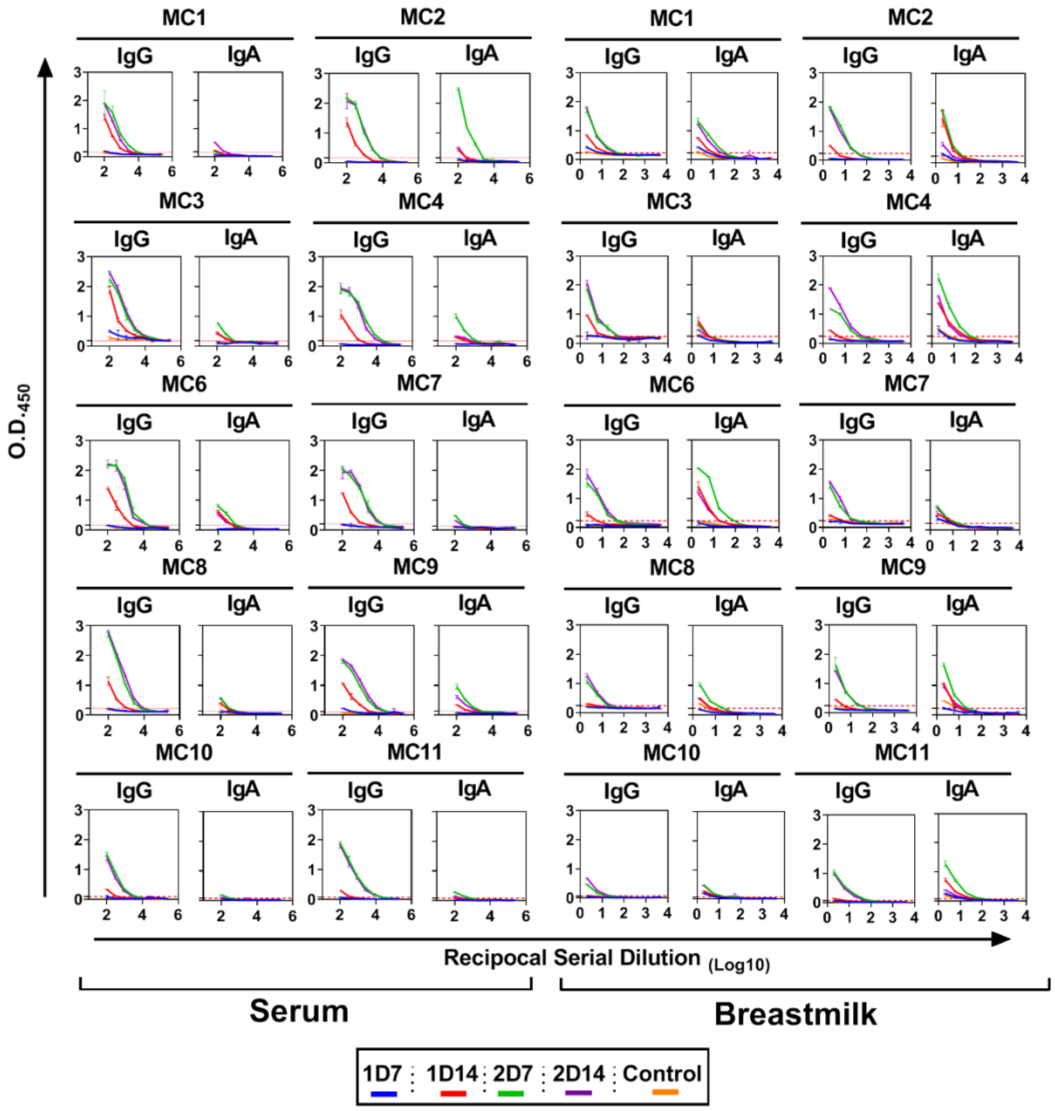

b

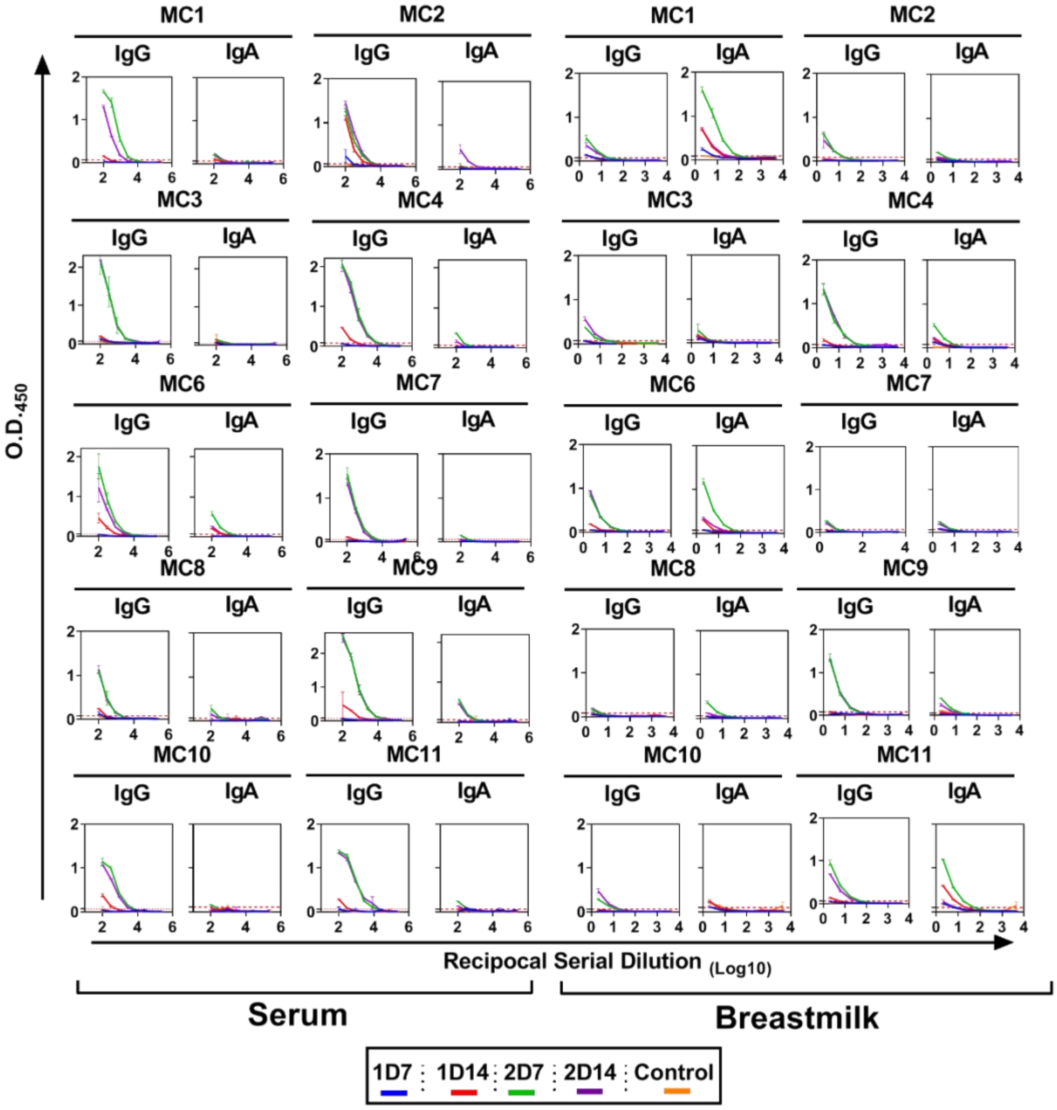
MC1
MC2
IgG
IgA
MC3
MC4
MC6
MC7
MC8
MC9
MC10
MC11
O.D.450
Recipocal Serial Dilution (Log10)
Serum
Breastmilk
1D7
1D14
2D7
2D14
Control

Supplementary Fig. 2 Temporal dynamics of spike and RBD specific antibody responses. Endpoint titers were calculated and plotted by time points following the first and second vaccine doses for each participant. Breastmilk and serum samples were tested against **a** SARS-CoV-2 spike, and **b** RBD protein. Y-axis units are endpoint titers on a linear scale.

a

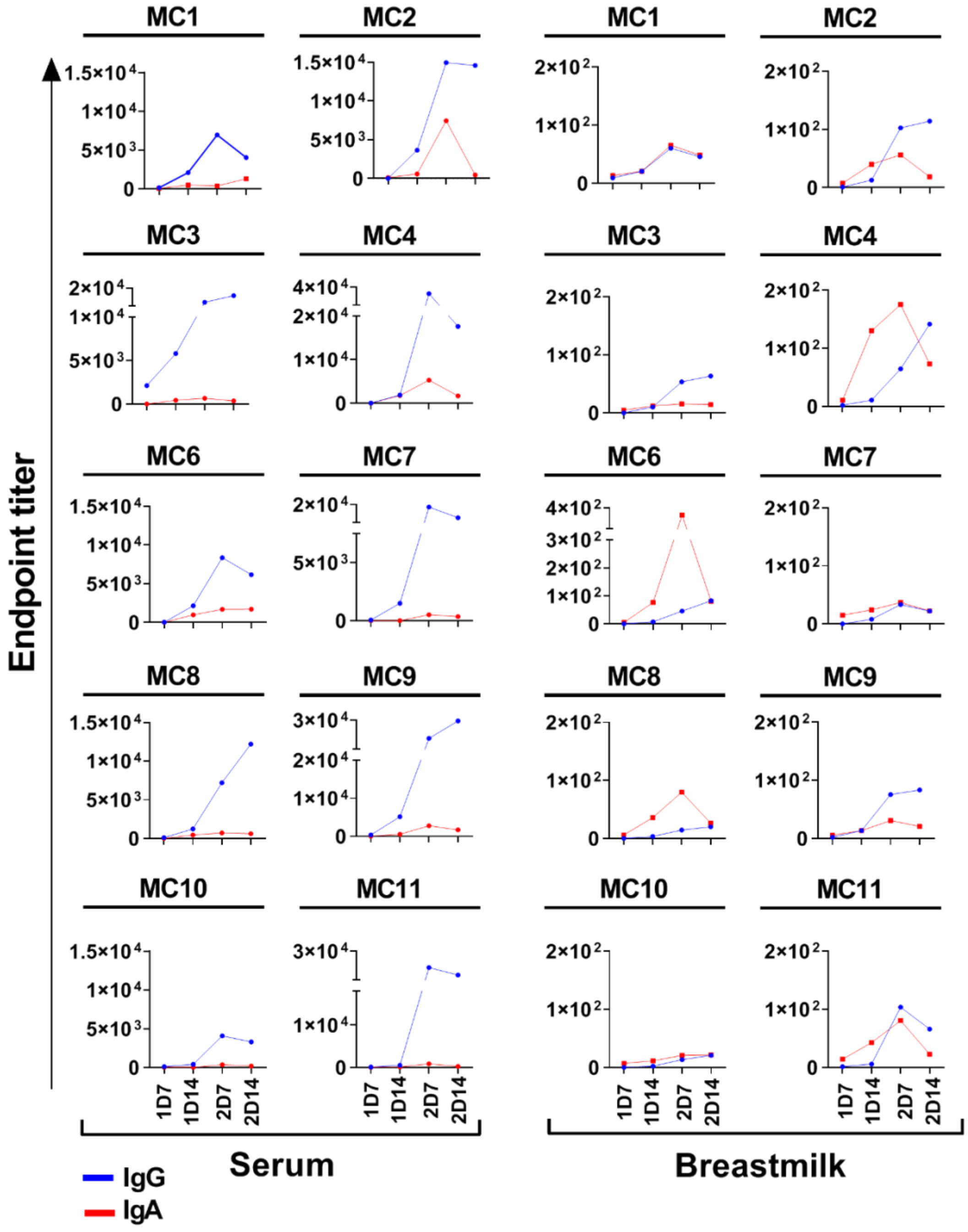

b

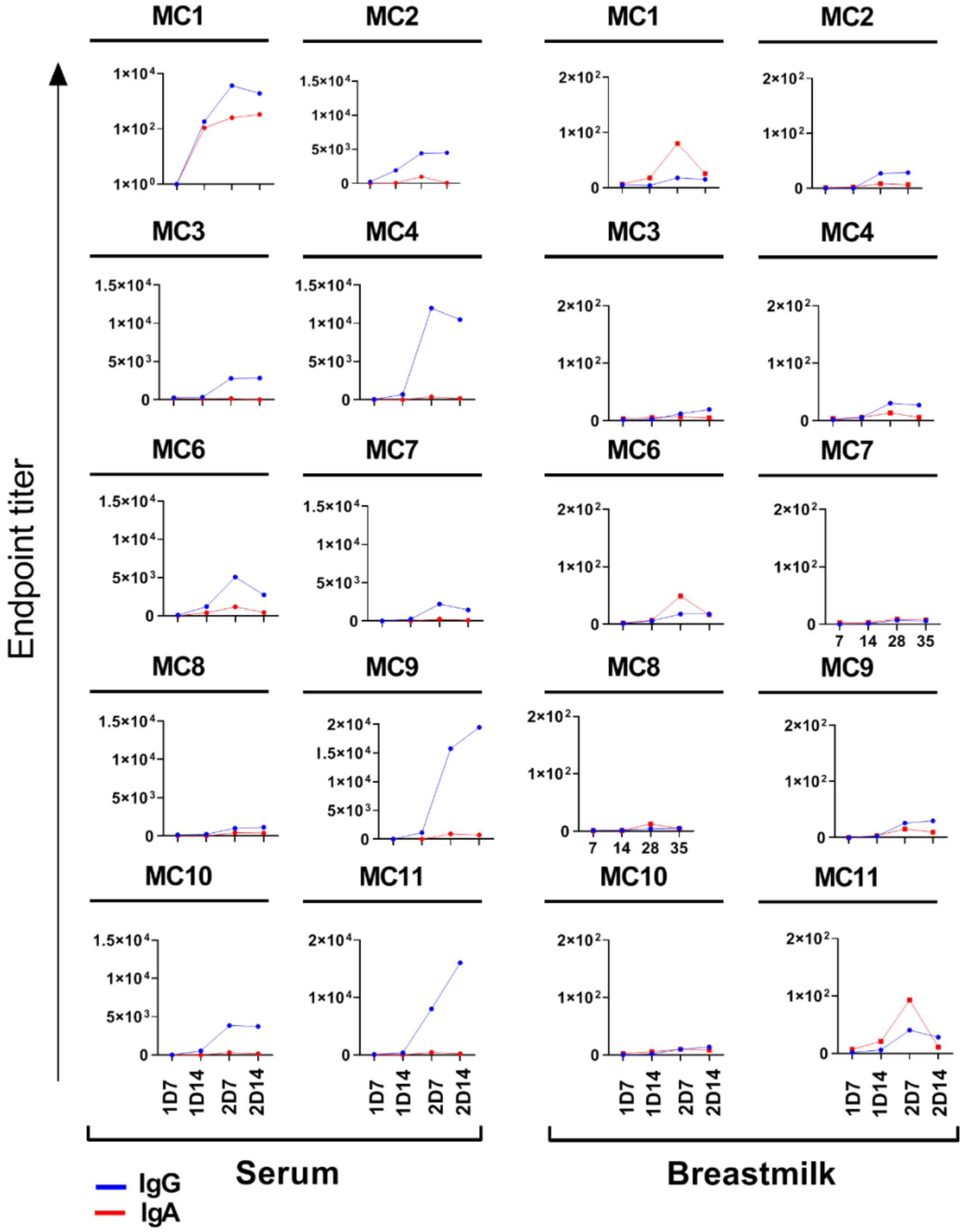

Endpoint titer
MC1
MC2
MC3
MC4
MC6
MC7
MC8
MC9
MC10
MC11
1D7
1D14
2D7
2D14
7
14
28
35
Serum
Breastmilk
IgG
IgA

Supplementary Fig. 3 Endpoint titers of RBD-specific antibodies in breastmilk and serum (n=10). a Comparison between the levels of vaccine-specific IgG and IgA antibodies as determined by ELISA endpoint titers. Endpoint titers were interpolated by applying a four-parameter logistic curve (4PL) on reciprocal dilution series for all serum and breastmilk samples, at four time points (by color), for IgG and IgA. P values were determined with an unpaired, two-sided Mann-Whitney U-test after applying Bonferroni correction; $P < 0.0125$ was considered statistically significant. Results are presented as geometric means and 95% confidence intervals. Y-axis units are endpoint titers on a logarithmic scale. Control serum (n=10) and breastmilk (n=10) samples were obtained prior to the COVID-19 pandemic. **b** Endpoint titers per participant. Each colored line represents the titers for each participant. Green and blue arrows indicate the time points of administration of the first (t=0) and second (t=21) doses of the mRNA vaccine, respectively. Y-axis units are endpoint titers on a linear scale.

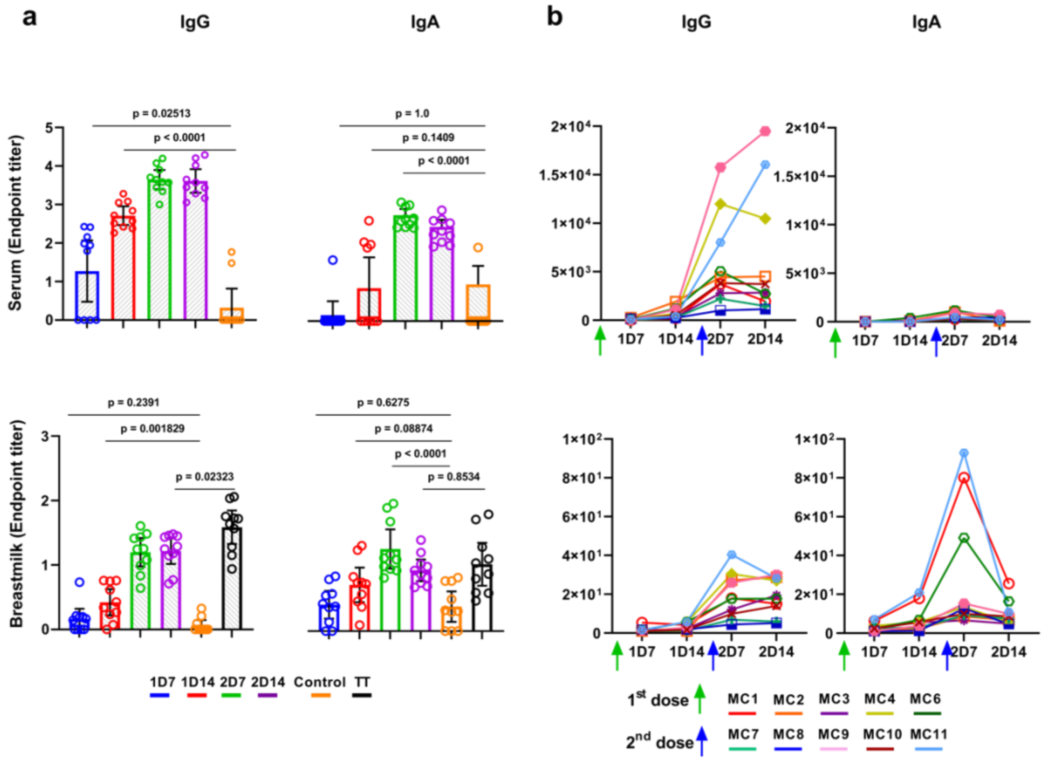

Supplementary Fig. 4 Spike-specific IgG:IgA molar ratio in breastmilk and serum at 4 time points following first and second doses of mRNA vaccine. a Comparison of IgG:IgA ratio in serum vs. breastmilk (n=10) were made using the two-tailed Wilcoxon signed-rank test; P < 0.05 was considered statistically significant. The IgG:IgA molar ratio in serum (n=10) **b,** and breastmilk (n=10) **c** is shown as bar plots and comparison between three time point (1D14, 2D7 and 2D14) and the first time point (1D7) was tested by applying non-parametric Friedman test and correcting for multiple comparison using Dunn's test. p values are shown. Median with 95% CI is indicated.

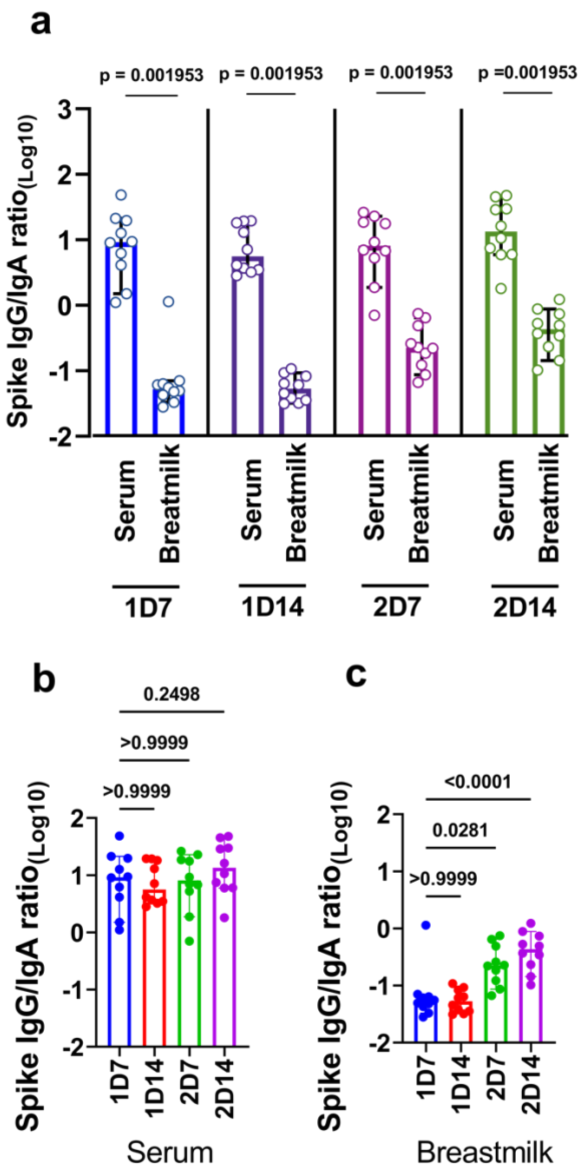

Supplementary Fig. 5 Dynamics of the RBD-specific antibody response. Fold-change in antibody titers compared to the first time point (1D7) are plotted by patient. Comparisons between the fold-change in RBD-specific IgG and IgA antibodies were performed using the two-sided Wilcoxon signed-rank test; P < 0.0125 following Bonferroni correction (accounting for multiple comparisons) was considered statistically significant. Y-axis units are fold-change on a logarithmic scale.

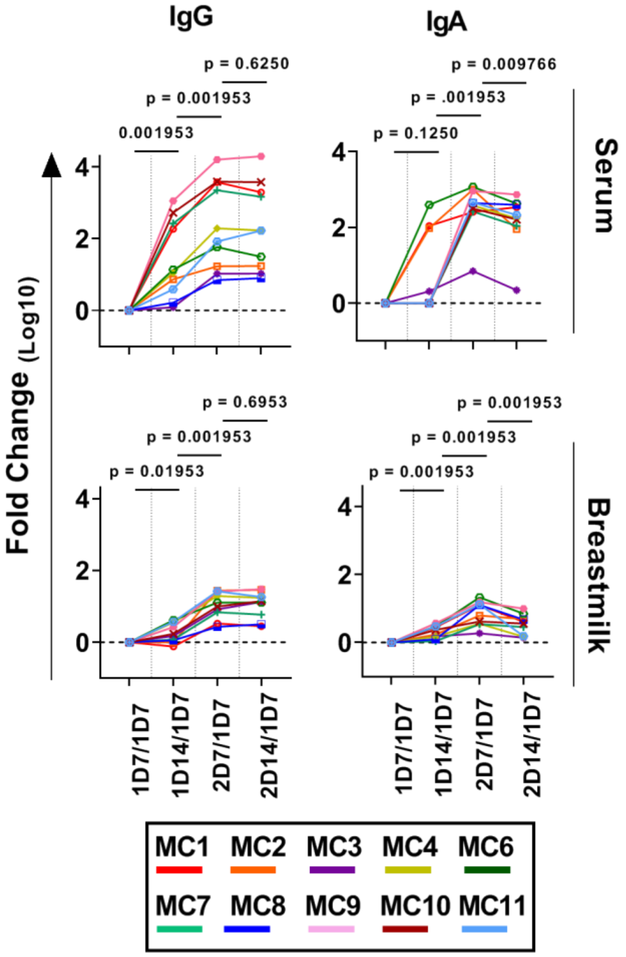

Supplementary Fig. 6 Fold-change of the endpoint vaccine-specific antibody titers. Line graphs showing the endpoint titer fold-change in comparison to the proceeding time point for **a** the spike, and **b** the RBD protein. Comparisons between the fold-change in vaccine-specific IgG and IgA antibodies were made using the two-sided Wilcoxon signed-rank test; P < 0.0125 following Bonferroni correction (accounting for multiple comparisons) was considered statistically significant. Y-axis units are fold-change on a logarithmic scale.

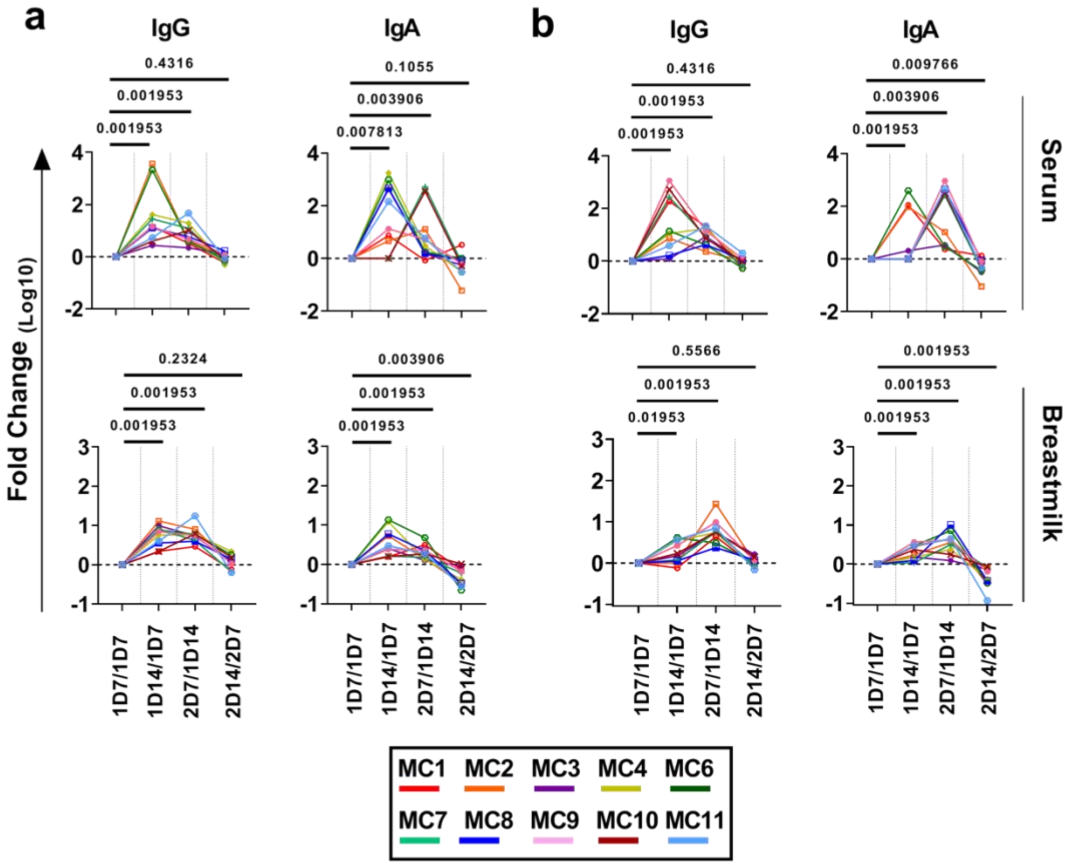

Supplementary Fig.7. XTT assay was performed on HEK293 ACE2-stable cell line treated with either 50 percentage of not filtered breastmilk or serial dilutions of filtered breastmilk ranging from 3.125 to 50 percentage of final volume. Untreated or 30% DMSO-treated cells served as negative and positive controls for cell toxicity, respectively. All conditions were conducted in triplicates. Error bars represent SEM. P values were determined with an unpaired, two-sided Mann-Whitney U-test; $P < 0.05$ was considered statistically significant. ns- non significant.

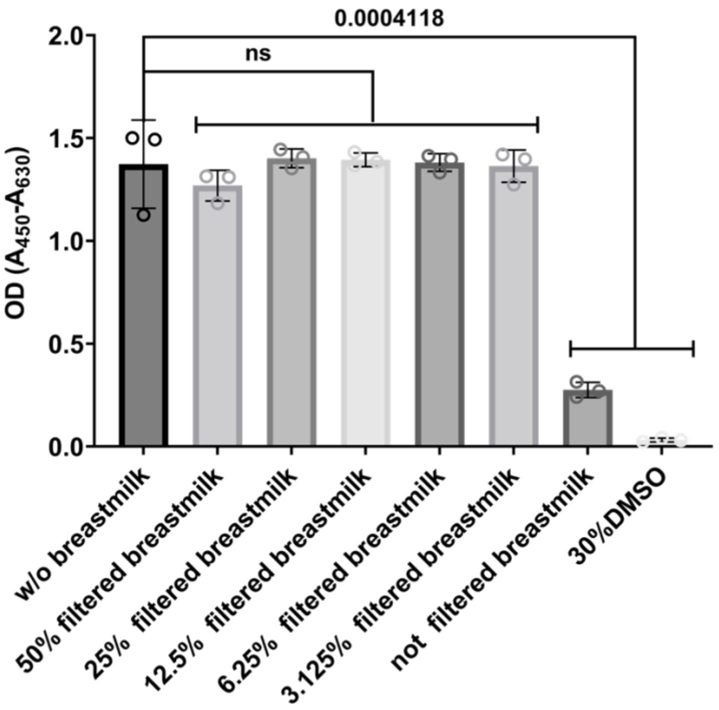

Supplement: Supplementary file 1 — Supplementary Information [file 41467_2021_26507_MOESM1_ESM.pdf]
