# Supplementary material for: BNT162b2 mRNA vaccine elicited antibody response in blood and milk of breastfeeding women
Source: Nat Commun. 2021 Oct 28;12:6222. doi: 10.1038/s41467-021-26507-1 (PMC8553805; doi:10.1038/s41467-021-26507-1)
Supplement: Supplementary file 3 — Reporting Summary [file 41467_2021_26507_MOESM3_ESM.pdf]

nature portfolio

Corresponding author(s): Yariv Wine

Last updated by author(s): Sep 23, 2021

Reporting Summary

Nature Portfolio wishes to improve the reproducibility of the work that we publish. This form provides structure for consistency and transparency in reporting. For further information on Nature Portfolio policies, see our Editorial Policies and the Editorial Policy Checklist.

Statistics

For all statistical analyses, confirm that the following items are present in the figure legend, table legend, main text, or Methods section.

n/a	Confirmed	
☐	☒	The exact sample size (*n*) for each experimental group/condition, given as a discrete number and unit of measurement
☐	☒	A statement on whether measurements were taken from distinct samples or whether the same sample was measured repeatedly
☐	☒	The statistical test(s) used AND whether they are one- or two-sided<br>*Only common tests should be described solely by name; describe more complex techniques in the Methods section.*
☐	☒	A description of all covariates tested
☐	☒	A description of any assumptions or corrections, such as tests of normality and adjustment for multiple comparisons
☐	☒	A full description of the statistical parameters including central tendency (e.g. means) or other basic estimates (e.g. regression coefficient) AND variation (e.g. standard deviation) or associated estimates of uncertainty (e.g. confidence intervals)
☐	☒	For null hypothesis testing, the test statistic (e.g. *F*, *t*, *r*) with confidence intervals, effect sizes, degrees of freedom and *P* value noted<br>*Give P values as exact values whenever suitable.*
☒	☐	For Bayesian analysis, information on the choice of priors and Markov chain Monte Carlo settings
☒	☐	For hierarchical and complex designs, identification of the appropriate level for tests and full reporting of outcomes
☒	☐	Estimates of effect sizes (e.g. Cohen's *d*, Pearson's *r*), indicating how they were calculated

Our web collection on statistics for biologists contains articles on many of the points above.

Software and code

Policy information about availability of computer code

Data collection	GraphPad Prism version 9.0.2
Data analysis	GraphPad Prism version 9.0.2<br>R version 4.1.1

For manuscripts utilizing custom algorithms or software that are central to the research but not yet described in published literature, software must be made available to editors and reviewers. We strongly encourage code deposition in a community repository (e.g. GitHub). See the Nature Portfolio guidelines for submitting code & software for further information.

Data

Policy information about availability of data

All manuscripts must include a data availability statement. This statement should provide the following information, where applicable:

- Accession codes, unique identifiers, or web links for publicly available datasets
- A description of any restrictions on data availability
- For clinical datasets or third party data, please ensure that the statement adheres to our policy

Data are available on request due to privacy or other restrictions

Field-specific reporting

Please select the one below that is the best fit for your research. If you are not sure, read the appropriate sections before making your selection.

- [x] Life sciences
- [ ] Behavioural & social sciences
- [ ] Ecological, evolutionary & environmental sciences

For a reference copy of the document with all sections, see nature.com/documents/nr-reporting-summary-flat.pdf

Life sciences study design

All studies must disclose on these points even when the disclosure is negative.

Sample size	10 healthcare providers and 10 for negative control. Blood and breastmilk samples.
Data exclusions	n/a
Replication	All measurement were carried out in duplicates or triplicates.
Randomization	n/a
Blinding	n/a

Reporting for specific materials, systems and methods

We require information from authors about some types of materials, experimental systems and methods used in many studies. Here, indicate whether each material, system or method listed is relevant to your study. If you are not sure if a list item applies to your research, read the appropriate section before selecting a response.

Materials & experimental systems

n/a	Involved in the study	
[ ]	[x]	Antibodies
[ ]	[x]	Eukaryotic cell lines
[x]	[ ]	Palaeontology and archaeology
[x]	[ ]	Animals and other organisms
[ ]	[x]	Human research participants
[ ]	[x]	Clinical data
[x]	[ ]	Dual use research of concern

Methods

n/a	Involved in the study	
[x]	[ ]	ChIP-seq
[x]	[ ]	Flow cytometry
[x]	[ ]	MRI-based neuroimaging

Antibodies

Antibodies used	Goat anti human polyclonal IgG (Jackson Immunoresearch, #CAT 109035003) and goat anti-human IgA, alpha chain specific (Jackson Immunoresearch, #CAT 109035011) both Horseradish Peroxidase Conjugated
Validation	The following list of citation provided by the manufacture utilized the anti human IgG (#CAT 109035003) :https://www.citeab.com/antibodies/2036588-109-035-003-peroxidase-affinipure-goat-anti-human-ig?utm_campaign=Widget+All+Citations&utm_medium=Widget&utm_source=Jackson+Immunoresearch&utm_term=Jackson+ImmunoResearch<br>The following list of citation provided by the manufacture utilized the anti human IgA(#CAT 109035011) : https://www.citeab.com/antibodies/2036591-109-035-011-peroxidase-affinipure-goat-anti-human-se ?utm_campaign=Widget+All+Citations&utm_medium=Widget&utm_source=Jackson+Immunoresearch&utm_term=Jackson+ImmunoResearch

Eukaryotic cell lines

Policy information about cell lines

Cell line source(s)	Expi293F GnTI cell line by ThermoFisher Scientific
Authentication	provided and authenticated by ThermoFisher Scientific
Mycoplasma contamination	N/A
Commonly misidentified lines (See ICLAC register)	N/A

Human research participants

Policy information about studies involving human research participants

Population characteristics	10 lactating, immunized, healthcare providers, mean age 34.6 (range 30-38)
Recruitment	The women were asked to participate in the study after recieving the first dose of Pfizer-BioNTech BNT162b2 COVID-19 mRNA vaccine
Ethics oversight	IRB number 0002269-4 and 0002757-1 given at Tel Aviv University and under ethical approval number 1088-20-TLV given at Tel Aviv Sourasky medical center

Note that full information on the approval of the study protocol must also be provided in the manuscript.

Clinical data

Policy information about clinical studies

All manuscripts should comply with the ICMJEguidelines for publication of clinical research and a completedCONSORT checklist must be included with all submissions.

Clinical trial registration	N/A
Study protocol	N/A
Data collection	N/A
Outcomes	N/A
